# Epistemic innocence and the production of false memory beliefs

**DOI:** 10.1007/s11098-018-1038-2

**Published:** 2018-01-19

**Authors:** Katherine Puddifoot, Lisa Bortolotti

**Affiliations:** 1 Department of Philosophy, University of Birmingham, Birmingham, UK

**Keywords:** Memory, Epistemic benefits, Adaptiveness, Epistemic innocence

## Abstract

Findings from the cognitive sciences suggest that the cognitive mechanisms responsible for some memory errors are adaptive, bringing benefits to the organism. In this paper we argue that the same cognitive mechanisms also bring a suite of significant *epistemic benefits*, increasing the chance of an agent obtaining epistemic goods like true belief and knowledge. This result provides a significant challenge to the folk conception of memory beliefs that are false, according to which they are a sign of cognitive frailty, indicating that a person is less reliable than others or their former self. Evidence of memory errors can undermine a person’s view of themselves as a competent epistemic agent, but we show that false memory beliefs can be the result of the ordinary operation of cognitive mechanisms found across the species, which bring substantial epistemic benefits. This challenge to the folk conception is not adequately captured by existing epistemological theories. However, it can be captured by the notion of epistemic innocence, which has previously been deployed to highlight how *beliefs* which have epistemic costs can also bring significant epistemic benefits. We therefore argue that the notion of epistemic innocence should be expanded so that it applies not just to beliefs but also to *cognitive mechanisms*.

## 1 Introduction

In this paper we focus on three types of false memory beliefs that have been identified within the cognitive science literature, and on the cognitive mechanisms that produce those mental representations. Our discussion focuses on false memory *beliefs* rather than false memories *simpliciter*. This is an important distinction because someone could have a true memory that x happened but have false memory beliefs about x. For example, a person with Alzheimer’s disease might correctly remember that an event happened, but falsely believe that it happened yesterday when it actually happened 30 years ago. There could be multiple cognitive mechanisms leading to the formation of any memory belief, including those leading to memory *simpliciter* and those that produce beliefs about the memory. The scope of our discussion includes all of these mechanisms because they all contribute to the production of memory beliefs and our aim is to show that there can be epistemic benefits associated with the possession of the cognitive mechanisms that lead to the production of the false memory beliefs.

One common example of the type of memory error that is discussed in the cognitive sciences is produced by *imagination inflation*: people who imagine an event that never occurred subsequently falsely recall that the event did occur. Another example is the *Deese*–*Roediger*–*McDermott* (*DRM*) *effect*: after having studied a list of words, people falsely believe that items were on the list that were absent but are semantically related to words that were on the list. The *post*-*information effect* is a further example: people seemingly remember details of an experienced event. They did experience the event (e.g., a car accident), but not the detail in question (e.g., the car bumping onto something), which they have learnt about from other sources at a later stage (e.g., a witness mentioned it in the police report) and incorporated into their memory belief.

Here we want to focus on the *benefits* associated with the production of the three types of false memory beliefs.^1^ The potential benefits of false memory beliefs have received some attention in the psychological literature, especially in Daniel Schacter’s work:

While it is tempting to conclude that memory distortions point to fundamental flaws in the nature or composition of memory, a growing number of researchers have argued that, to the contrary, many memory distortions reflect the operation of adaptive processes – that is, processes that contribute to the efficient functioning of memory, but as a consequence of serving that role, also produce distortions. (Schacter et al. 2011, page 467)

In previous discussions, researchers have tended to focus on the *psychological* or *biological* benefits associated with certain types of false memory beliefs (see for instance Boyer 2008; Sutton 2009; Fernández 2015), that is, on how certain types of false memory belief can improve the agent’s wellbeing and good functioning, or increase her genetic fitness in some environment. More recently, some *epistemic* benefits of have also been associated with false memory beliefs (see for instance, McCarroll 2017^2^; Bortolotti and Sullivan-Bissett forthcoming). The focus in the latter project is to identify ways in which false memory beliefs are associated with features of human cognition that improve the chance of an agent achieving epistemic goals, including acquiring new true beliefs; retaining and using relevant information; increasing the coherence of a set of beliefs; and gaining understanding.^3^ Our main task in this paper is to establish that there are frequent, systematic and varied *epistemic* benefits associated with the *operation of the cognitive mechanisms* that are implicated in the production of certain types of false memory beliefs. Moreover, these epistemic benefits occur in the absence of any epistemic benefits of specific memory beliefs.

In particular, we identify a class of cases in which agents have cognitive mechanisms that produce false memory beliefs, and the mechanisms could easily be characterised in terms of the epistemic costs associated with the memory beliefs. For our purposes in this paper, we adopt a broad understanding of ‘cognitive mechanism’. A cognitive mechanism is a mental operation that processes information and typically produces beliefs or belief-like states. We emphasise that, although the cognitive mechanisms we examine in this paper systematically and predictably produce false memory beliefs, they also facilitate the achievement of a suite of important epistemic goals, including goals other than the formation of accurate memory beliefs. In other words, some memory beliefs that solely or overwhelmingly bring epistemic costs are the result of the ordinary operation of cognitive mechanisms that are epistemically beneficial due to their contribution to various aspects of epistemic agency. We conclude that, due to the valuable epistemic contribution of the cognitive mechanisms, and the loss that would be incurred in the absence of the ordinary operation of the mechanisms, it is useful to acknowledge the epistemic benefits of such mechanisms as well as their costs. Our view provides a significant challenge to folk conceptions of memory errors, according to which they provide an indication of cognitive frailty. We argue that existing epistemological theories fail to reflect the epistemic benefits that we identify. For example, a reliabilist account might evaluate the reliability of the cognitive mechanisms that produce false memory beliefs on the basis of whether they reliably produce true memory beliefs. It might find that the memory mechanisms tend to produce true memory beliefs or that they tend to produce false memory beliefs. Either way, the approach would fail to capture the breadth of the phenomenon we identify: cognitive mechanisms that contribute to the achievement of *various* epistemic goals, including goals other than the formation of true memory beliefs. In contrast, an expanded and suitably modified notion of epistemic innocence can be used to tag the benefits of those mechanisms. We take this to show that the notion of epistemic innocence, that was previously deployed to capture the epistemic benefits associated with epistemically costly *beliefs* can also be usefully deployed to capture the epistemic benefits of the possession of epistemically costly *cognitive mechanisms*.

The structure of the paper is as follows. First, we show that some types of false memory beliefs that are commonly discussed in the cognitive sciences standardly bring no substantial epistemic benefits themselves but are produced by cognitive mechanisms that bring substantial epistemic benefits. To achieve this goal, we discuss each of the three types of false memory belief already introduced in this section (DRM illusion, imagination inflation, post-event information effect). There are epistemic costs associated with each of the illusions, but there are also significant epistemic benefits to the possession of the mechanisms responsible for those illusions, and the benefits of the mechanisms can occur in the absence of any benefits of the memory beliefs. We then argue that existing epistemological theories cannot capture this important and robust phenomenon. Finally, we show how an expanded notion of epistemic innocence can usefully achieve this goal.

## 2 The Deese–Roediger–McDermott illusion

This section begins the project of showing that there while there are epistemic costs associated with the possession of cognitive mechanisms that produce specific false memory beliefs there are also significant epistemic benefits. A feature of human cognition is epistemically beneficial if it improves the chance of an epistemic agent forming true beliefs or achieving some other epistemic goal. On the other hand, it is epistemically costly if it reduces the chance of an epistemic agent forming true beliefs or achieving some other epistemic goal. Let us begin, then, by considering how the cognitive mechanisms responsible for the *Deese*–*Roediger*–*McDermott* (DRM) illusion can be viewed as epistemically costly but also bringing significant epistemic benefits. To achieve this goal we first need to provide more details about the phenomenon.

### 2.1 The Phenomenon of the DRM illusion

In the DRM experimental paradigm (developed by Deese 1959, and revised by Roediger and McDermott 1995), participants are presented with a list of words to study, e.g. *baker, butter, filling, brown, dough, grain, flour, knife, wheat, old*, and then they are asked to recall the items on the list. Participants systematically and predictably claim that there were words on the list which were not there but are semantically related to words that are on the list: this phenomenon has become known as the *DRM illusion*. For example, those who studied the list just presented frequently recalled having studied the word *bread* that was absent from the list.^4^ Asked about whether they could mentally re-experience studying the words or merely knew that they had studied such words, participants indicated that they could mentally experience studying the words, although they had not previously studied them.^5^ Questions are often raised about whether phenomena discovered in the lab are also found in a naturalistic setting, but in this case there are studies suggesting that evidence of false recall on DRM is correlated with memory distortions found outside the experimental setting (Gallo 2010). For example, participants with higher level of false recall on the DRM are more likely than controls to provide inaccurate details about where they were when they heard about an important event (e.g., the O.J. Simpson trial) if they were asked a few months after the event (Platt et al. 1998). Thus, there is reason to think that individuals who are subject to the DRM illusion are also susceptible to forming false memory beliefs due to the operation of the effect out of the lab.

### 2.2 Epistemic costs of the DRM illusion

The DRM illusion brings epistemic costs. A person who is subject to the illusion inaccurately represents reality and lacks self-knowledge. Take Sylvia who is shown the list of words mentioned above: *baker, butter, filling, brown, dough, grain, flour, knife, wheat*, and *old*. She makes the common error of believing that the word *bread* was on the list. She positively affirms that she can re-experience seeing the word rather than merely knowing that she saw it. She has a false memory and misrepresents the past. The false memory leads her to have a false belief about the words that she actually studied.

### 2.3 Epistemic benefits of cognitive mechanisms underpinning the DRM illusion

In a standard case we can suppose that the person who is subject to the DRM illusion gains no further epistemic benefits as a result of forming this false memory belief. Sylvia does not gain any advantage as a result of falsely believing that bread was on the list. However, the cognitive science literature suggests that there are significant advantages associated with the possession of the cognitive mechanisms responsible for the DRM illusion.

[A]ssociative processes provide structure and organization that aids memory performance, and gist-based processes support retention of themes and meanings that facilitate generalization and abstraction. (Schacter et al. 2011, p. 469)

Given the best explanations of the DRM illusion, the cognitive mechanism has an adaptive function. In this section we show that on the same explanations the mechanisms also facilitate the achievement of some significant epistemic goals.

According to one explanation, when participants read (or hear) a list of words, they automatically think of other words with associated meanings. So, to return to the earlier example, when the words *baker, butter, filling, brown, dough, grain, flour, knife, wheat*, and *old* are read (or heard), the concept *bread* is also activated. The activation of the associated concept is responsible for people claiming that they can “re-experience” the event of studying the word. What they re-experience, on this view, is the concept being activated.^6^ On this explanation, the DRM illusion is a by-product of an adaptive feature of human cognition, namely the associative nature of cognitive structures that facilitate “inferences” (Roediger and McDermott 2000).

When it is argued that associative mechanisms facilitate inferences, it is not meant that they facilitate *careful inferential reasoning*. Instead, it is meant that the mechanisms lead to the triggering of thoughts that are semantically related to items that are encountered in thought or action. The ordinary operation of associative processes can be extremely useful in navigating the environment. Items become associated with each other through associative learning, via exposure to items that are spatio-temporally co-located. As a result, the associations often reflect environmental regularities. The cognitive systems underpinning memory lead to the triggering of thoughts about items related to target items that have been encountered, facilitating predictions about items that are likely to be found in an environment and the correct identification of items that are likely to be present.

This point can be illustrated by considering Schacter et al.’s (2011) discussion of *contextual associations*. Contextual associations are associations between items that typically occur together in a context. The following experiment measured contextual associations. Experimental participants occupied an office for 10 min and were then required to recall the items in the office. It was found that they systematically falsely recalled items that they had not encountered but which are typically found in an office setting (Brewer and Treyens 1981). The participants displayed the DRM illusion seemingly as a result of making an association between items that typically co-occur in an office context.

It can be epistemically useful to make contextual associations. If you are in a new workplace, for example, associating the items that you encounter with other items that you are likely to encounter, because they are frequently found in offices, will enable you to predict what you are likely to find in the novel environment. Making such contextual associations will often lead you to *successfully predict features of a new environment* and *adopt accurate beliefs about items likely to be found in that environment*. The activation of the contextual associations can therefore positively contribute to the achievement of epistemic goals, facilitating successful predictions and accurate beliefs about the environment, which count as significant epistemic achievements. If the DRM illusion is underpinned by the ordinary operation of associative mechanisms, it is the result of the operation of cognitive mechanisms that facilitate the achievement of significant epistemic goals.

A second explanation of the DRM effect is that people form *gist* representations of the items that they study as well as verbatim representations with the exact details of items on the list (Brainerd and Reyna 2002). The gist representation summarizes the common theme found in the list. For example, when presented with the words *baker, butter, filling, brown, dough, grain, flour, knife, wheat, old*, people form a gist representation, for example, *bread*-*related items*. Distortions occur when people attempt to fill out the details of the list on the basis of the gist-representation. They list items that fit the common theme found in the list, including also items that were not on the original list. At this point, a person might, for example, take the gist representation *bread*-*related items* and infer, consciously or unconsciously, that bread was included in the list.^7^

Gist-based processing is credited with numerous benefits. It enables memory systems with only limited storage capacity to preserve relevant information economically (Schacter et al. 2007), in compact event records (Schacter et al. 2011). As details fade, the compact, abstract, gist-based representation (e.g. bread-related items) remains. The information that remains can be used in abstract thinking, inference, and convergent thinking. Imagine, for example, that Antonia has been handed a shopping list by her partner and asked to pick up the items from the shop on the way home from work. On the list are butter, eggs, baking powder and flour. Antonia forms an abstract “gist” representation of the list: *ingredients for a cake*. On the basis of this abstract representation, she concludes that her partner plans to bake a cake for after dinner. Antonia accidentally leaves the shopping list at work and ends up going shopping without it. She might falsely recall that some cake ingredients (e.g. sugar) were on the list when they actually were not. Nonetheless, as a result of possessing the cognitive mechanism underlying gist-based recall, she will have gained the benefits of being able to remember that her partner wanted her to buy cake ingredients even after the verbatim record of ingredients has faded from her memory. She can infer that her partner is going to bake a cake and that she needs to get butter, eggs, baking powder and flour, and make other useful inferences on the basis of the gist representation.

What this example illustrates is that if the DRM illusion is the result of the formation of a gist-based representation, then it is underpinned by a cognitive mechanism that has significant benefits, in terms of enabling *retention of key information* after the memory of specific items or events has faded, enabling *inferences from known to unknown facts*, and supporting *the making of broad associations* which is a component of creativity. All of these benefits have implications for the achievement of epistemic goals. The acquisition and retention of key information to guide thought and action is an epistemic goal. The ability to make an inference from known to unknown facts is an epistemic achievement, enabling new true beliefs to be formed on the basis of existing knowledge. Meanwhile the ability to draw broad connections between different things that are known can be crucial to enabling humans to engage in inference that can produce new knowledge. As the capacity to make gist representations enhances the pursuit of these goals it therefore brings significant epistemic benefits.

Under both dominant explanations, thus, the DRM illusion is the result of the ordinary operation of cognitive mechanisms that can systematically and predictably produce false memory beliefs, but have an important role to play in the achievement of some significant epistemic goals, including goals other than remembering. In other words, the cognitive mechanisms have significant epistemic benefits. These benefits can occur in the absence of benefits of the specific false memory beliefs, as can be seen in the case of Antonia. There is no benefit to falsely remembering that sugar was on the shopping list but there was nonetheless a significant epistemic benefit to the possession of the cognitive mechanism leading her to believe that it was there.

## 3 Imagination inflation

### 3.1 The phenomenon of imagination inflation

Let us now consider imagination inflation. In studies of this effect, participants who are asked to imagine an event that never occurred are more likely than participants who are not asked to imagine the event to (a) increase their confidence that the event occurred, (b) believe that they were somehow involved with the event (e.g. as a witness or protagonist), and (c) have a full blown episode of recall of the event.^8^ Cases of imagination inflation can involve false memory belief, where a person falsely recalls that an event occurred in their past as a result of imagining the event.^9^

### 3.2 Epistemic costs of imagination inflation

There are substantial epistemic costs associated with imagination inflation. When people believe that they experienced an event, or details of an event, that they only imagined, they (a) suffer a failure of self-knowledge, failing to know what they actually experienced, and (b) become disposed not to respond appropriately to the available evidence about what they have actually experienced, taking what is imagined to provide evidence in support of a belief (that event x happened, or happened in way y) rather than basing their belief on experiential evidence. They become disposed to form further false beliefs that are consistent with the false memory produced as a result of imagination inflation.

Consider Jei who comes to believe that she was left in a shopping mall as a child although this event was just something she imagined. Not only does Jei have false beliefs about the past (“I was left in a shopping mall as a child”) and lack self-knowledge, but she is also likely to form further beliefs that are consistent with the false memory belief and equally fail to reflect reality. For instance, Jei might come to believe that the parent looking after her at the time must have felt really panicked and guilty.

### 3.3 Epistemic benefits of cognitive mechanisms underpinning imagination inflation

In a standard case of imagination inflation there will not be any epistemic benefit to believing that something occurred when it was only imagined. However, the dominant existing explanations of the phenomenon suggest that it is underwritten by cognitive mechanisms that subserve important epistemic goals. According to this explanation, imagination inflation is the result of a combination of factors: (1) some memory systems are recruited for both remembering the past and imagining the future (Schacter et al. 2007); (2) the nature of memory is reconstructive (Schacter et al. 2007); (3) source-monitoring is required to distinguish remembering the past from imagining the future (Garry et al. 1996). We will suggest that the close links between imagination and memory and the reconstructive nature of memory (1) and (2) also facilitate the achievement of important epistemic goals.

The explanation of imagination inflation goes as follows. There is a core or “default” network of cognitive systems that are recruited for both imagining the future and remembering the past.^10^ These systems are activated when a person imagines an event as well as when they remember the past. In both cases, information is stored in the “default” network. There is a source monitoring system that functions to tag the source of the information, e.g. personal experience, testimony, or imagination. However, when imagination inflation occurs the source monitoring system fails. A person imagines that an event occurred. The information is stored in the “default network”. There is a source monitoring error. Due to the common underpinnings of memory and imagination, confusion between remembering and imagining occurs. Information that was only imagined is tagged as information from past experience. The imagining is misidentified.

In addition to this, information from memory and imagination can become combined because, according to the *constructive simulation* hypothesis, when we remember or imagine we rely not on complete records of discreet events but on traces of information. Such traces of information from memory and imagination are stored in the same cognitive systems. When traces of information are retrieved, they are combined, to represent an event that either happened in the past or could happen in the future (Schacter et al. 2007). When the memory system constructs memories from traces, it can combine traces of information about past events with traces of information stored as a result of imagination, resulting in imagination inflation. Take, for example, Gloria’s thought, *I met Tom in the park this time last year*, which seems to be a recollection of a past event but is actually the result of imagination inflation. In fact Gloria met another friend, Tyra, in the park. Around the same time, Gloria imagined meeting Tom who she has not seen for some time. Traces of information about Gloria’s meeting with Tyra are stored in the same cognitive system as traces of information about her imagined meeting with Tom. The traces get combined in a way that fails to accurately represent the past event, because memory constructs rather than replicates the past.

It is now possible to understand how there are epistemic benefits associated with the possession of the cognitive mechanisms responsible for imagination inflation. There are benefits in having the capacity to combine traces of information in different ways. Such a capacity enables agents to make predictions about novel future events, facilitating flexible predictions about the future that draw on a diversity of information about the past (Schacter et al. 2007). As the future does not always resemble the past, the flexibility of thought offered by such a cognitive system will often be instrumental to making accurate predictions. The close link between memory and imagination facilitates predictions about the future because memories of past episodes provide information about what might happen in the future, and imagination uses the information to simulate the future (Boyer 2008). As it is an important epistemic goal to form true beliefs about the future, it can be epistemically beneficial for memory and imagination to be so closely linked.

In sum, then, the best existing current explanations of imagination inflation suggest that some of the cognitive mechanisms underlying the phenomenon facilitate the achievement of various epistemic goals.^11^ They enable people to *successfully predict the future* by thinking flexibly and to draw on a variety of sources of information. It is valuable that imagination and memory are so closely linked because memory provides an important contribution to the process of imagining what will happen in the future.

## 4 Post-event information effect

### 4.1 The phenomenon of post-event information effect

The *post*-*event information* effect occurs when information encountered after an event influences people’s recollection of that event (Loftus 1996). One common source of this information is other people’s testimony. The beliefs produced by the post-event information effect present a case of false memory belief even when the information provided after the event is accurate, because the person believes that *they can remember* details of an event that they do not remember. They only think that they remember those details due to being exposed, after the event, to further information.

A type of post-event information effect is the *misinformation* effect, which occurs when *false* information about an event influences the memory belief. The misinformation effect gives rise to a false memory belief, because it leads people to import false information about an event into a memory belief, compromising the accuracy of such belief. For example, Loftus and Pickrell (1995) asked participants to think and write about four events that supposedly occurred in their childhood. Three of the four events actually happened: the information about the events was accurate and had been supplied by a family member. The fourth event had not actually happened; each participant was told that she had been lost in a shopping mall as a child but she had not been. Six out of twenty-four participants falsely remembered the false event when subsequently tested. In other studies, participants were shown a clip of an event, for example, a simulated car crash (e.g. Loftus and Palmer 1974). Afterwards, some of the participants were given additional, false information about the event that they had just seen in the clip. Participants who were given the false information were more likely than the others to have false memory beliefs about the event (e.g., falsely recalling that there was broken glass where there was none, or falsely believing that a clean-shaven man had a moustache instead).

### 4.2 Epistemic costs of post-event information effect

There are substantial epistemic costs to the post-event information effect. People who falsely believe that they remember details of an event consequently lack self-knowledge. Consider Arjun who is asked about what he remembers from a film he went to see at the cinema yesterday. Arjun provides information that comes from his friends’ recount of the film and not from his own experience of it, but he sincerely claims to *remember* the information he provides.^12^ Because Arjun is asked about what he remembers, the relevant evidence consists in the information he collected through his own experience of the film. By presenting information from testimony as something that he remembers, Arjun reports a belief that is poorly supported by the evidence available to him.

The misinformation effect has been the object of intense debate among psychologists and legal theorists because eyewitnesses and claimants in criminal trials can be susceptible to the effect (for a comprehensive overview of the vast literature see Zaragoza et al. 2007). For example, suggestive police questioning can lead to eyewitnesses believing that they remember certain things about a crime when they do not. In such a case, the eyewitness forms a belief about the crime that is poorly supported by their experience of the crime, instead reflecting information presented to them after the experience. They lack self-knowledge about what they experience. As a result, they can provide false testimonial evidence.

In general, people who are subject to the post-event information effect respond inappropriately to the evidence that they have available to them, treating the testimony provided by other people after an event as if it is memorial evidence of the event. Assuming that different weight should be given to these different sorts of evidence (testimonial and memorial), the person who misidentifies the former with the latter will make an epistemic error. The situation is of course even worse from an epistemic perspective if the person has been provided with inaccurate testimonial evidence: not only will they be likely to form false beliefs about the details of an event, but they are unlikely to recognise that they have depended upon unreliable testimony because they will believe that they remember the details.

### 4.3 Epistemic benefits of cognitive mechanisms underpinning post-event information effect

Now let us consider the best existing explanation of the post-event information effect found in the cognitive sciences, and how it suggests that there are epistemic benefits associated with the presence of the cognitive mechanisms underpinning the effect. Recent work in neuroscience suggests that the misinformation effect is the product of a process called *reconsolidation* (Hupbach et al. 2007; Schacter et al. 2011). When information is retrieved from memory systems, and the information is reactivated, the memory that is retrieved is in a labile state, requiring a period of stabilization, during which new information can be incorporated (Nader and Einarsson 2010). There is a window of opportunity for the brain to alter the memory. Memories can be strengthened or weakened in this process, and the contents of memories can be updated, with new information incorporated. Reconsolidation explains the misinformation effect in the following way: when a memory relating to an event is reactivated and false information relating to that memory is available, the false information can be incorporated into the memory during the period of reconsolidation (Hupbach et al. 2007; Schacter et al. 2011).

The mechanism of reconsolidation can therefore bring the epistemic costs outlined in 3.2. but it can also bring substantial epistemic benefits. The process of reconsolidation facilitates the formation of memories that reflect the most accurate, up-to-date information. It can thus facilitate the retention of accurate information and the possession of true beliefs. Take a case in which a Johnnie has studied a book about butterflies and later watched a television programme on the same subject. A few months later Johnnie does not remember watching the television programme. He does recall that the book contained some specific fascinating information. In fact, the information was not presented in the book but in the television programme. What has happened is that while Johnnie was watching the television programme, his memory of reading the book was reactivated, there was a period in which the memory was in a labile state requiring a period of stabilization. In this window of opportunity the memory belief about reading the book about butterflies is updated to reflect the information contained in the television programme. The memory belief that the information in the book is false but it is nonetheless epistemically beneficial that Johnnie has a mechanism that updates the memory of reading the book because it enables him to recall the fascinating information, to identify butterflies correctly, and so on, even though his memory of watching the television programme has faded.

One of the uses of the tendency to update memory beliefs to reflect information encountered after an event is *epistemic vigilance*; the capacity of an agent to track the reliability of other agents (Sperber et al. 2010). Memory beliefs are an important source of information about other people—facilitating the prediction and explanation of other people’s behavior within a particular setting by enabling recall of past episodes that are similar in some respect (Boyer 2008). The ability to combine stored information about past events with newly gathered information, changing how one recalls an event to reflect new information, allows the events stored in memory to reflect the most recent available information (ibid.).

For example, you encounter a new colleague, Josh, in the hallway at work. He might avoid eye contact and not say “hello”. Your memory belief about this event can provide an important source of information about Josh, and, in particular, about whether he is going to be a co-operative informant about events in your workplace. However, the information that you gather during this event is limited. Later, you talk about the meeting in the hallway with another colleague, Rosa, and Rosa informs you that Josh has just found out that his father has a serious illness. The event is still going to strongly influence your judgement about Josh and your predictions about the success of future interactions with him, because you depend upon recall of past episodes to make predictions about the future. It will therefore be beneficial for your impression of the event to reflect the most up-to-date information available to you. One way that your memory systems can facilitate this is by leading you to recall the event differently from how you initially experienced it; you might now recall that Josh seemed to you distracted or distressed. Now you recall the event in a way that better reflects the most up-to-date information that you have rather than your initial impression. Future assessments and predictions of Josh’s conduct, for example, about whether he will supply information that you need for your work, can draw on your memory belief about the event while also reflecting what you found out about Josh after the event.

Given that testimony is so central to our epistemic lives, the proposed benefit of the post-event information effect is a substantial epistemic benefit. The phenomenon of people depending on others as sources of information, via testimony, is ubiquitous. As agents frequently depend on others as a source of information, they need to be able to track who is and who is not a reliable and co-operative communicator (Craig 1990; Sperber et al. 2010). If the post-event information effect allows us to make assessments of others that better track how reliable and co-operative they are, then it will bring substantial epistemic benefits. It will increase the chance that we identify trustworthy people to depend upon as sources of testimony.

There is another interesting connection between the post-event information effect and testimony. Not only does the post-event information effect have the potential to improve testimonial exchanges and more generally allows us to base decisions and judgements on more up-to-date information, but its contribution to the achievement of epistemic goals depends upon successful testimonial exchanges. This is because the epistemic benefits associated with the effect depend on the accuracy of the information provided after the event and testimony is a common source of this information (Michaelian 2013).

Those focusing on the empirical literature on the post-event information effect could be forgiven for thinking that the phenomenon more often than not leads to errors in judgements and predictions because misleading information about an event influences memory. However, this impression would be an artifact of the experimental design. When measuring the post-event information effect, participants are often provided with misleading information about an event, and then tested to see whether their judgements reflect the misleading information (see, e.g. Loftus and Pickrell 1995). This experimental design has the advantage of being able to identify those memory beliefs that are influenced by the information presented after the event: those memory beliefs will reflect the false information. The observation that a majority of studies on the post-event information effect focus on cases in which misleading information influences memory does not therefore provide good reason for thinking that in most cases inaccurate information is used to update memory beliefs.

In contrast, recent research on testimony and lying provides reason for thinking that the information provided to an individual via testimony after an event is likely to be accurate (Michaelian 2013). It suggests that people only tend to tell lies when they speak about themselves (De Paulo and colleagues 1996).^13^ People tend to tell the truth as a default, choosing to lie only when telling the truth would hinder the their goals, for example, leading to some degree of social awkwardness, tension, or discomfort (Levine et al. 1999).^14^ This means that as long as people sharing information are knowledgeable about the subject matter that they are discussing, and are not talking about themselves, or aiming to achieve some goal that they cannot achieve through truth-telling, they are likely to present accurate information. If this information is integrated with other information deriving from a memory belief, then details that might have been missed or misrepresented can be added to the memory belief and used to improve future judgement and decision-making.

There can be epistemic benefits, then, to possessing the cognitive mechanism underlying the post-event information effect. Reconsolidation enables memory beliefs to be updated to reflect the most recent *accurate* information. People are often reliable sources of information so accurate information from testimony will often be available to update existing memory beliefs. But where people are not reliable sources of information reconsolidation will facilitate the identification of this fact due to its supporting epistemic vigilance by enabling memory beliefs about the reliability of other people to be updated to reflect the most recent accurate information. The cognitive mechanisms underlying the post-event information effect can therefore facilitate the important epistemic goals of storing the most recent information and identifying others who are reliable sources of information.^15^

## 5 A robust but unacknowledged phenomenon

Sections 2–4 have identified a robust psychological phenomenon: cognitive mechanisms that systematically and predictably produce false memory beliefs but also bring significant epistemic benefits. This is an important result. It challenges the intuitive view of memory errors and provides reason for adopting a new, more forgiving approach towards memory errors. It is deeply intuitive that memory errors are a sign of the frailty of an individual’s cognitive capacities, showing that a person who makes an error is more likely than others, or their past self, to make other errors.^16^ When we mistakenly remember that x occurred or that x occurred in way y we might take this to be a sign that we are becoming more susceptible to making errors than our past selves and others. We might consequently view ourselves as incompetent epistemic agents.

In contrast, the discussion so far shows that some memory errors are the result of the ordinary operation of cognitive mechanisms that are found across the human species. They do not indicate that memory systems are becoming more susceptible to errors. It might nonetheless be natural to think that it would be altogether preferable from an *epistemic* perspective to have memory systems that function like a storehouse from which we could retrieve complete and accurate records of discreet events. The arguments presented in Sects. 2–4 show that this too is a mistake. There are epistemic benefits associated with the possession of cognitive mechanisms that store information in forms other than complete and accurate records of discreet events. The arguments of Sects. 2–4 therefore present a challenge to folk conceptions of human memory errors.

Existing epistemological theories are not able to adequately capture this challenge. A brief survey illustrates this point. Deontological theories focus on whether or not a believer has done all that she could do with respect to the goal of forming a true belief (see, e.g. Chisholm 1977). Virtue responsibilist theories focus on whether the believer has displayed positive intellectual character traits in the formation of the belief, e.g. being conscientious, open-minded, etc. (Montmarquet 1987, 1993; Zagzebski 1996). On each of these types of accounts, a belief has good epistemic standing if the epistemic agent has acted in a responsible way, doing their duty or being virtuous. Because of the focus on the responsibility of the epistemic agent, these epistemological accounts are not able to capture the epistemic benefits of cognitive mechanisms responsible for the formation of memory belief distortions. When cognitive mechanisms lead a person to think about associated concepts, form gist-representations, combine information from imagination and memory and update memory beliefs these phenomena are not the result of a believer being epistemically responsible. They are not the result of intentional action on the part of the believer. The epistemic benefits of the operation of the cognitive mechanisms will consequently not be captured by the deontological or virtue responsibilist approaches.

It might seem as if the shortcoming of the deontological and virtue-responsibilist approaches is that they focus on the epistemic agent rather than the cognitive mechanisms that systematically and predictably lead to the formation of false memory beliefs. It might therefore be thought that an approach that focuses on cognitive mechanisms will be more successful. The reliabilist approach is representative of such an approach. On a reliabilist approach, positive epistemic standing is attributed to a belief if a reliable belief forming process, cognitive mechanism or method produces it (see, e.g. Goldman 1979). It might be thought the reliabilist approach can capture the epistemic benefits of the cognitive mechanisms that produce false memory beliefs by tagging those mechanisms as reliable (Michaelian 2011, 2013). However, there are a number of reasons why a reliabilist approach would fail to capture the robust phenomenon identified in Sects. 2–4.

First, reasons have been given in Sects. 2–4 for thinking that the cognitive mechanisms bring various epistemic benefits. But this does not mean the mechanisms reliably produce true beliefs. Indeed, we have stressed that the same cognitive mechanisms that bring epistemic benefits often produce false memory beliefs and remained neutral about whether the ratio of true to false beliefs means that it is appropriate to label the mechanisms as reliable.

Second, on a reliabilist approach, memory beliefs would be accorded positive epistemic status only if they were produced by belief forming processes, cognitive mechanisms or methods that reliably produce true *memory beliefs*. The reliabilist approach could therefore capture the phenomenon of false memory beliefs being formed by mechanisms that reliably produce true memory beliefs. But this is not the nature of the phenomenon under current discussion. The current discussion focuses on how false memory beliefs can be formed by mechanisms that improve the chances of the believer achieving numerous epistemic goals, including future-oriented goals like predicting the future and identifying reliable testifiers who will provide a good source of information in the future. A reliabilist approach would fail to capture the breadth of epistemic benefits that can be gained as a result of the operation of the cognitive mechanisms that produce distorted memory beliefs by solely focusing on the tendency of the mechanisms to produce true *memory* beliefs.

A consequentialist approach that assigns positive epistemic status to a belief based on whether the possession of the belief brings positive epistemic consequences (see, e.g. Bortolotti 2015, 2016) also fails to capture the types of cases outlined in Sects. 2–4. According to such an approach, a false memory belief could be accorded positive epistemic status as long as it increased the chances of the believer obtaining some epistemic good, such as true belief, knowledge or understanding. However, the focus of the current discussion is on the epistemic benefits of possessing the cognitive mechanisms responsible for false memory beliefs, not the benefits of possessing the beliefs. It is therefore necessary to switch attention away from the epistemic benefits of the beliefs towards the epistemic benefits of the cognitive mechanisms to properly focus on the phenomenon identified in Sects. 2–4.

There is therefore an important and robust phenomenon of false memory beliefs being produced by the ordinary operation of cognitive mechanisms that bring various epistemic benefits, improving our chances of obtaining epistemic goods like true belief, knowledge and understanding. This phenomenon challenges the folk conception of memory errors as solely indicating that a person is less reliable than their past self or others, e.g. as a source of information. The folk conception suggests that our memory errors should present a challenge to our view of ourselves as competent epistemic agents. This, in turn, can influence how we view our intelligence, our potential to contribute to public discourse, and so on. It would therefore be of great value to be able to tag the phenomenon identified in Sects. 2–4. However, existing epistemological theories do not adequately capture the phenomenon.

## 6 Introducing epistemic innocence

So far, then, it has been found that there is a robust psychological phenomenon that could challenge the folk conception of memory errors and that epistemological theories do not capture this phenomenon. This section introduces the notion of *epistemic innocence*, which, it shall be argued in the next section, can capture the phenomenon if it is suitably expanded.

The notion of *innocence* is deployed in a variety of contexts, but there are two senses of ‘innocent’ that resonate with the use we propose and both come from the legal account of innocence-defence (see Botterell 2009). In so-called *justification-defence*, an act that is objectionable is not condemned when it prevents serious harm from occurring. Innocence here is due to the act being an effective response to an emergency situation. In legal contexts, self-defence is the most common example of this form of innocence. The person is not criminally liable for acting in self-defence even though her act would in other circumstances constitute an offence. In so-called *excuse*-*defence*, an act that is objectionable is not condemned when the person performing it either could not have done otherwise (e.g., as in duress or compulsion) or did not realise that the act was objectionable (e.g., due to intoxication or insanity). Innocence here is due to the person not being responsible for performing the act.^17^

It is useful to apply the notion of *innocence defence* to the epistemic domain. We can think about whether adopting a belief that is inaccurate or ill-grounded has any epistemic benefits. For instance, having an epistemically costly belief is epistemically innocent iff:

(*Epistemic Benefit*) Adopting the belief delivers some significant epistemic benefit to an agent at a time, such as the prevention of a serious epistemic harm; and

(*No Alternatives*) A less epistemically costly belief that would deliver the same epistemic benefit is not available to that agent at that time.

The notion of epistemic innocence has been applied so far to instances of beliefs that have evident epistemic costs but also significant epistemic benefits that could not be obtained in the absence of the costs. Examples of epistemically innocent beliefs are motivated delusions (Bortolotti 2015), delusions in schizophrenia (Bortolotti 2016), delusions in depression (Antrobus and Bortolotti 2016), confabulated explanations of actions driven by implicit bias (Sullivan-Bissett 2015), mental states resulting from the use of psychedelic drugs (Letheby 2016), inaccurate social cognitions that represent the world as more equal than it is (Puddifoot 2017), and distorted memories in the clinical population (Bortolotti and Sullivan-Bissett forthcoming).

Why does epistemic innocence matter? The philosophical literature on the epistemic evaluation of beliefs (e.g., is this belief worth having?) and on the so-called ‘ethics of belief’ (e.g., are agents blameworthy for adopting a false or irrational belief?) has been dominated by the issues of whether a belief accurately represents reality and whether it is sufficiently supported by evidence. Other possible contributions of beliefs to epistemic agency have been neglected, probably because they are often difficult to measure. However, even a false or irrational belief, one that would not meet the standards of accuracy and justification, can play an important role in the achievement of epistemic goals. For instance, in some contexts, the presence of that belief could encourage the agent to look further rather than stopping a search for relevant information or to share information with others, thereby making feedback available to the agent. The point of the notion of epistemic innocence as applied to beliefs is to make room for these valuable epistemic contributions that false or irrational beliefs can make despite failing to achieve accuracy or justification.

Thus, the notion of epistemic innocence, as it has been deployed so far, is useful because it captures the contributions to epistemic agency that false or irrational beliefs can make. In particular, it captures the fact that, due to the features of the context in which the belief is adopted, its epistemic contributions are unique to the given belief: it would be difficult or impossible for a less epistemically costly belief to make the same valuable contribution. Obviously, to claim that a belief is epistemically innocent is not to claim that it is epistemically *good* overall, or epistemically *justified*. For one thing, one may find after a cost–benefit analysis that the epistemic costs of the belief still outweigh its epistemic benefits. To recap, to say that a belief is epistemically innocent is to indicate that it has epistemic benefits that would be either difficult or impossible to attain otherwise, and thus its epistemic contribution cannot be dismissed just because it is false or irrational.

## 7 Epistemic innocence and false memory beliefs

The notion of epistemic innocence is therefore valuable because it can tag the epistemic value of beliefs that are inaccurate or irrational. It might therefore seem as if the false memory beliefs produced by the DRM, imagination inflation and the post-event information effect can be identified as epistemically innocent; that the notion of justification-defence and excuse-defence can be usefully applied to them. Some false memory beliefs in the clinical context seem to be good candidates for epistemic innocence (Bortolotti and Sullivan-Bissett forthcoming). In the context of advanced dementia, inaccurate memory beliefs can be conceived as a way to retain some accurate information about the self that could otherwise be lost. Moreover, they can contribute to the person maintaining social contact which enables her to exchange information and receive feedback from her peers.

In contrast, the characterisation of the notion of epistemic innocence applied so far to delusional beliefs, confabulated explanations and other epistemically costly beliefs does not capture the case where a type of belief has no epistemic benefit itself, but there are significant epistemic benefit in the mechanism(s) responsible for the production of that type of belief. It therefore does not capture the types of cases identified in Sects. 2–4. However, an expanded notion of epistemic innocence can achieve this goal. For this reason, we believe that extending the scope of the epistemic innocence notion is worthwhile. In particular, we argue that the notion of epistemic innocence can usefully be deployed to capture the phenomenon identified in Sects. 2–4 if it is extended, and suitably modified, to apply to *cognitive mechanisms* as well as individual beliefs. By extending the notion of epistemic innocence in this way it is possible to capture how we should epistemically value false memory beliefs, despite their epistemic costs, not because they also have epistemic benefits, but because they are the result of cognitive mechanisms that make a significant contribution to the achievement of important epistemic goals.

On the proposed view, a cognitive mechanism is epistemically innocent when it meets the three following conditions.

- (*Epistemic Cost*) Cognitive mechanism produces epistemically costly cognitive states.
- (*Epistemic Benefit*) Cognitive mechanism has some *significant* epistemic benefits for an agent.
- (*No Alternatives*) There is no available alternative cognitive mechanism that would enable the agent to avoid the epistemic costs while conferring the same epistemic benefits.

It has already been shown that the Epistemic Cost and Epistemic Benefit conditions are met by the cognitive mechanisms that produce the DRM illusion, imagination inflation and the post-event information effect. The mechanisms bring epistemic costs because they lead to false memory beliefs, a lack of self-knowledge, and false beliefs downstream (Sects. 2.2, 3.2, 4.2). It has also already been shown that they bring epistemic benefits in various forms (Sects. 2.3, 3.3, 4.3).

It is therefore important to focus on whether the cognitive mechanisms meet the *No Alternatives* condition. It will be suggested here that it is unlikely that there are cognitive mechanisms that enable the agent to avoid the epistemic costs of having false memory beliefs while bringing the epistemic benefits of the cognitive mechanisms that produce the DRM, imagination inflation, and post-information effect. A cognitive mechanism of this type would produce true memory beliefs under the conditions in which the DRM effect, imagination inflation and the post-event information effect would lead to false memory beliefs.^18^ They would do this while facilitating associative thinking, abstract thinking and retention of key information, successful flexible and dynamic prediction of the future, and the formation of memory beliefs reflecting the most recent information available to the agent. Given the speculative nature of this issue, the availability of alternative cognitive mechanisms, here we will just provide some reasons to support the view that it is unlikely that there are cognitive mechanisms that meet each of these conditions.

The main point to be recognised is that while it is within the space of logical possibilities that there could be cognitive mechanisms that produce true memory beliefs where the DRM illusion, imagination inflation and the post-event information effect lead to false memory beliefs, evidence from the cognitive sciences strongly suggests that this is not within the space of *psychological* possibilities. A memory system could store complete and accurate representations of what happened in the past. It could, in other words, function like a storehouse, storing accurate files of information ready for retrieval at some later point. However, there is consensus within the cognitive sciences (see, e.g. Schacter et al. 2007, 2011) and philosophy (see e.g. De Brigard 2014; Sutton 1998, 2010; Michaelian 2011; Robins 2016), and it is increasingly being acknowledge in neuroscience (see, e.g. Stickgold and Walker 2013; Eichenbaum and Cohen 2014; Richards and Frankland 2017) that this is not how memory systems work. It is not that there are cognitive systems available to an epistemic agent or some epistemic agents at any point in time that could produce accurate memory beliefs from a storehouse. Instead, humans are solely equipped with *reconstructive memory systems* that store traces of information about events that can be flexibly recombined but also systematically and predictably produce false memory beliefs.^19^

The above point is nicely illustrated by considering how the DRM illusion is explained in terms of gist-representations. If we had memory systems that worked like a storehouse we could store an accurate and complete record of information about a list of words. But we do not have memory systems that operate in this way. Instead, on the gist-based explanation of the effect we briefly form verbatim representations of the items on the list, but this information quickly fades. What remains is a gist representation. Then the epistemic agent who wishes to remember what is on the list is required to construct a representation of the list based on the gist representation. It is not therefore possible for the agent to avoid the epistemic costs associated with having false memory beliefs by drawing upon a stored accurate and complete record of the list because such a record does not exist.

It might be possible to gain some of the epistemic benefits we have identified in Sects. 2.3, 3.3 and 4.3 from other cognitive mechanisms that do not systematically produce false memory beliefs. It is possible that some of the functions of the mechanisms we discussed could be played by a slower mechanism, that could allow for effortful reasoning and possibly be more effective at detecting distortions. However, there are two considerations that mean that such a mechanism would not present a relevant alternative.

First of all, such a mechanism would only bring the epistemic benefits under a restricted set of circumstances. It would not enable us to gain the benefits under conditions of high cognitive load or limited time. The trade off between speed, automaticity, and parsimony on one side, and accuracy and conscious control on the other side, is a well-established subject of debate in cognitive science (see, e.g. Kahneman’s 2011). What this means is that people will *not* be able to gain the epistemic benefits without the epistemic costs in many contexts, i.e. those in which speedy, automatic and parsimonious responses are required. In contrast, the flexible and dynamic memory mechanisms implicated in the memory errors we discuss are likely to be able to function under conditions of high cognitive load and time limitations to produce epistemic benefits like predicting the future.

Second of all, a slower cognitive mechanism that allows for effortful reasoning would not obviously directly produce memory beliefs. Recall that an alternative to the mechanisms that we are suggesting are epistemically innocent would be a mechanism that *produced true memory beliefs* under the conditions where the DRM illusion, imagination inflation and the post-event information effect produce false memory beliefs (while also bringing the relevant epistemic benefits). But if we engage in careful inference, thereby gaining the epistemic benefits of forming broad associations, engaging in abstract thinking, and making inferences about what might happen in the future, including inferences that reflect the most up-to-date information that we have available to us, the product of these inferences is not likely to be a memory belief. We might remember making an inference and remember the product of the inference (e.g. remembering having a belief about what will happen in the future) but the memory beliefs would be an indirect by-product of the inference. A cognitive mechanism that produces an inferential belief (e.g. about the future) will not present a relevant alternative to the cognitive mechanisms that produce false memory beliefs, in the sense that is important to the current discussion, if it does not produce true memory beliefs because it does not (directly) produce any memory beliefs.

In sum, then, although it is logically possible for there to be cognitive mechanisms available to the epistemic agent that avoid the epistemic costs of false memory beliefs, producing true memory beliefs where the DRM illusion, imagination inflation and the post-event information effect produce false beliefs, humans are solely endowed with reconstructive memory systems. These systems produce false memory beliefs as a part of the process of bringing significant epistemic benefits. Other cognitive mechanisms might bring some of the same epistemic benefits through a process of careful inferential reasoning that could detect errors. However, these benefits will only be gained in some contexts; not those in which people are required to respond flexibly and efficiently to information found in their environments (due to cognitive load, exhaustion, etc.). In contrast, the memory systems that produce false memory beliefs are flexible and efficient and can bring benefits even under such unfavourable conditions. Moreover, cognitive mechanisms that utilise careful inferential reasoning to gain benefits such as making broad associations between information, engaging in abstract reasoning and predicting the future are likely not to directly produce memory beliefs. They will therefore not ensure that the agent avoids the epistemic costs associated with false memory beliefs in the relevant sense because they will not produce true memory beliefs.

What this means is that the notion of epistemic innocence, extended to apply to cognitive mechanisms, can capture the phenomenon of false memory beliefs being formed by cognitive mechanisms that bring a vast set of epistemic benefits. The cognitive mechanisms are epistemically innocent because although their operation brings epistemic costs by producing false memory beliefs it also has significant epistemic benefits for an agent. Moreover, no alternative cognitive mechanism would avoid the costs to the agent that are associated with false memory beliefs while conferring the same epistemic benefits.

With this extended notion of epistemic innocence it is possible to tag the cognitive mechanisms that produce the types of false memory beliefs that are discussed here and commonly in cognitive science in a way that captures how there can be epistemic gains associated with the production of the false memory beliefs. It is possible to identify the error that people make when they assume that their memory errors indicate that they are poor epistemic agents. It is possible to articulate the valuable roles performed by the cognitive mechanisms that systematically and predictably produce false memory beliefs that are not captured by focusing on the accuracy of the memory beliefs alone.

## 8 Conclusion

Memory errors bring substantial epistemic costs. However, we have shown that the cognitive mechanisms that produce false memory beliefs commonly discussed in the cognitive sciences often produce a variety of significant epistemic benefits. They can facilitate the achievement of significant epistemic goals, such as the formation of broad associations between information, the acquisition of knowledge gained through abstract thinking, and successful predictions about the future that reflect the most up-to-date information available to the agent.

It is natural to take memory errors to indicate the cognitive frailty of the person making the errors, showing that they less reliable as a source of information than others or their past self, and even that they are a poor epistemic agent. However, we have shown that memory errors can be the result of the ordinary operation of cognitive mechanisms found across the species that bring great epistemic benefits.

Neither folk psychology nor existing epistemological theories can adequately capture the epistemic benefits of the possession of the cognitive mechanisms that produce the false memory beliefs discussed in this paper. However, the notion of epistemic innocence, suitably extended and modified to apply to cognitive mechanisms, can do so. This means that the notion of epistemic innocence can contribute to an improved understanding of a crucial aspect of our cognitive and epistemic lives.

## Footnotes

1 Some beliefs are based on memories that are inaccurate in some respect but have a kernel of truth. Other beliefs are based on memories that are wholly fabricated. For example, a woman could falsely remember that she met her father in a café on the High Street when she actually met him on South Street. Her belief contains some accurate information as she met her father in a café. Alternatively, she could falsely remember that she met her father in a café on the High Street when she never met her father. Her belief does not contain any accurate information. Under some circumstances it is important to distinguish these two types of memory beliefs. For our current purposes, however, this distinction is not important. We shall discuss both types of memory belief under the rubric of false memory belief.

2 In the case of McCarroll (2017) things are actually a little more complicated. McCarroll argues that a particular type of memory, observer memories, which are commonly classified as distorted memories are not truly distorted and have epistemic benefits. Nonetheless, McCarroll argues that some mental states that are commonly taken to be distorted memories (see, e.g. Fernández 2015) have epistemic benefits.

3 The list of legitimate epistemic goals is not exhaustive here.

4 Participants presented with six lists falsely recalled studying words that are semantically related to the list words 40% of the time. This is the same rate at which participants recalled words that were present, but located in the middle rather than at the beginning or the end of the list. When the number of lists was increased to sixteen, and the number of words in the lists increased slightly, participants falsely recalled unstudied words 55% of the time.

5 In a follow-up study, Payne et al. (1996) replicated Roediger and McDermott’s results with a 24-hour gap between exposure to the lists and recall.

6 There is an important question regarding whether the DRM illusion captures semantic or episodic memory. Because the task requires recalling items from a list it seems to appeal to semantic memory, however, participants who claim to be able to “mentally re-experience” viewing words could be interpreted as appealing to episodic memory. The explanation of the DRM illusion in terms of associative processing provides reason to doubt that episodic memory underlies participants’ responses. The associative processing explanation suggests that participants who report being able to “mentally re-experience” studying an unstudied word are actually recalling the activation of a concept. The recollection of the activation of the concept does not amount to a recollection of an incident in a person’s life so does not seem to involve episodic memory. Due to concerns of this type our discussion focuses on the epistemic benefits of the mechanisms responsible for producing the DRM illusion while remaining neutral about the type of memory that is involved. Thanks to an anonymous reviewer for help with this point.

7 On the gist-based explanation of the DRM illusion it is more difficult than on the associative explanation to explain why participants claim to be able to “mentally re-experience” studying an unstudied word. This could be viewed as a point in favour of the associative view. However, it has been found that it is possible induce false memories that have a vivid phenomenology, that are characterised as “mentally re-experienced”, by repeatedly presenting different words with the same meaning (Reyna et al. 2016). This suggests that gist representations can be implicated in the production of false memories that involve being able to mentally re-experience; through strengthening the gist representation it is possible to induce participants to falsely believe that they can “mentally re-experience” a word. Further work is required to identify precisely how this process works. One plausible explanation of what happens when a person falsely remembers items to be on the list, that is fitting with the gist-based explanation, is that participants who claim to be able to “re-experience” studying an unstudied word recall the experience of filling out the details of the gist representation. Under such conditions it is inappropriate to say that the participants recall an experience of reading the unstudied word. For this reason, we might deny that the participant has a false episodic memory of studying the word. However, the participant would nonetheless have a false memory belief; a false semantic memory belief about the words that were on the list. Thanks to an anonymous reviewer for help with this point.

8 Interestingly, imagining that one was involved in an event that never happened is just as likely to produce false recall of the event as seeing a doctored video of the event in which one appears (Nash et al. 2009).

9 Not all cases of imagination inflation lead to false memory beliefs. In some scenarios participants increased their confidence in the likelihood that an event occurred but not to the level at which it would be proper to say that they believed that the event occurred. Our focus is on cases where people’s confidence increased to the point that it is appropriate to say that they had a belief that the event occurred.

10 The claim that the cognitive systems that are recruited for memory are also recruited for imagining the future is supported by fMRi studies showing common neural activation in remembering and imagining (e.g. Gonsalves et al. 2004; Kensinger and Schacter 2005; Schacter et al. 2012). In addition to this, it has been found that deficits in imagining often coincide with deficits in remembering (Kennett and Matthews 2009).

11 The source monitoring error that contributes to imagination inflation lacks systematic and predictable epistemic benefits. This does not detract from the importance of recognizing the epistemic benefits of the other features of human cognition that lead to the effect.

12 A study undertaken by Edelson et al. (2011) highlights the plausibility of this example. They presented participants with a film, which they watched with other people who they thought were their peers. The participants then underwent initial testing to discover what they remembered. Then, seven days after seeing the film, participants were presented with a task to test what they remembered, but this time some participants were also given false information about the film, purportedly provided by the other people who watched the film with them. Participants who were given the false information claimed to remember features of the film described in the information but not actually contained in the film.

13 The study results are rather bleak, suggesting that lying is an everyday occurrence. College students lied in one in three of their social interactions, and other members of the community, who were not college students, lied in one out of five of their social interactions. Community members lied on average once a day while college students lied twice a day. However, while these results are bleak, they suggest that in the majority of social interactions, people do not intentionally tell untruths.

14 Participants completed a forced choice activity, and were asked to state how they would respond to various situations that they might face in everyday life. In 62.5% of situations in which people had a motive to deceive in order to achieve some goal, they said they would deceive. In contrast, in only 1.6% of cases in which there was no motive to deceive did the participant say that they would engage in deception.

15 Of course reconsolidation can also bring epistemic costs, see Sect. 4.2 on the costs of its putative contribution to the misinformation effect. Our claim is not that the mechanism is infallible, or even that it is reliable, but rather that it can, at times, provide an important contribution to the achievement of epistemic goals, that it is useful to acknowledge.

16 In fact, the folk conception is probably a little more nuanced than this. It seems that we frequently allow that members of some groups (e.g. eccentric philosophy professors) can make errors without being cognitively frail. However, in general memory errors are taken to be a sign of cognitive frailty. Moreover, with regards to certain vulnerable social groups (e.g. older people, people with learning disabilities) memory errors are especially likely to be taken to be a sign of cognitive frailty. Our argument shows that this will frequently be an error.

17 According to Andrew Botterell (2009), the distinction between the two senses of innocence is controversial in the legal literature. Those who believe that there is a genuine distinction between the two talk about the person’s responsibility for the action or about her entitlement to act. In one version of the distinction, a justification seems to provide a stronger defence than an excuse: the person whose act is justified was entitled to act in that way, whereas the person whose act is excused is merely not blameworthy for performing it. The distinction might equally be understood this way in the epistemic context, though we do not discuss that here.

18 A cognitive mechanism could avoid the epistemic costs associated with producing false memories by producing no memories at all. However, a person endowed with such a mechanism would obviously be seriously hampered if they only used the mechanism, because they would have no memories. We are therefore concerned with whether the epistemic costs associated with false memory beliefs co-exist with the epistemic benefits associated with the cognitive mechanisms producing the memory beliefs.

19 We can sometimes use props, such as shopping lists or CCTV cameras, to ensure that we can access accurate information about events in the past. However, on many occasions we will not have access to any props (e.g. because we are not aware we will need them). As a result of the inconsistency of our access to these props, even when they are coupled with our internal cognitive systems they do not count as alternatives to our constructive memory system. On very many occasions coupled systems will not bring the epistemic benefits associated with reconstructive memory systems while avoiding the epistemic costs. On those occasions there will be no alternatives to the cognitive mechanisms that produce the false memory beliefs.
